# School Medical Service: Strategies to Promote Psycho-Physiological Well-Being

**DOI:** 10.3390/pediatric16010019

**Published:** 2024-03-19

**Authors:** Francesco Tafuri, Francesca Latino

**Affiliations:** 1 Heracle Lab Research in Educational Neuroscience, Niccolò Cusano University, 00166 Rome, Italy; francesco.tafuri@unicusano.it; 2 Department of Human Science, Educational and Sport, Pegaso University, 80100 Naples, Italy

**Keywords:** public medicine, pediatric medicine, physical activity, psychology, physiology

## Abstract

Schools represent the ideal setting for educating children about the acquisition of active lifestyles seen not only from a health point of view but also from psycho-pedagogical and social perspectives. Based on evidence from scientific literature, there is a need to include physical activity in school routines, especially in primary schools, where the habits learned by children stay with them in their later years and adulthood. With the support of the school medicine service, schools become a favorable context for planning health education sessions aimed at students, with particular reference to prevention. Within teaching, it is necessary to consider the motor area as a fundamental tool for acquiring correct lifestyles, facilitating cognitive development, inclusiveness, and psycho-emotional and socio-relational factors. Schools can play a fundamental role, becoming the key to promoting physical activity at different times of the day, such as during class hours (with active breaks), during breaks, before and after lessons, and by integrating movement into teaching. This review is the result of an in-depth overview of the available literature on the relationship of schools with health and health promotion from a preventive perspective, with awareness of how the issue is being approached and the need for further future reflections that will go hand in hand with the coming changes.

## 1. Introduction

Childhood represents an evolutionary phase of human existence that is decisive for all subsequent development. What is sown in this period of life determines, in part, the subsequent structure and balance of a person [1]. Schools, primary schools in particular, play a central role in promoting the practice of medical–psycho-physiological assistance services and the prevention of neuropsychological disorders in children [2]. Given the extreme urgency—typical of today’s society—to resolve various situations related to the medical–psycho-physiological assistance of children at the developmental age, the Italian School Medical Service offers a concrete response to the protection of the psychophysical health of school-aged pupils (from kindergarten to the end of secondary school) [3]. The role of the school medical service in disease prevention and psychophysical health promotion activities for pupils in compulsory schools refers to a multidisciplinary context that must consider the transversal needs and skills of children [4]. This is understood through a logic according to which the concept of health does not refer exclusively to the individual but is placed in a broader context that considers the collective well-being of people as a result of the quality of the environment and living spaces they find themselves in. Therefore, in response to citizens’ needs, the One Health approach requires the strengthening of a structured and coordinated network that must be reinterpreted according to the objectives of the National Recovery and Resilience Plan [5]. In this case, a set of strategies aimed at coping with social and health changes were carried out within a given region. They require a strategic and participatory approach that includes the school doctor, school principal, teachers, pupils, and families and facilitates consideration of the different current needs and knowledge on how to concretely approach the immediate challenges that present themselves. This is made possible by starting from the pivotal point of how schools—a fundamental setting for the promotion and protection of health—are considered by the National Prevention Plan 2020–25 [6].

An important starting point that pushes us to pay more attention to a renewed model of a school medical service oriented toward the promotion of health and the protagonism of all subjects involved (starting with very young students) can be found in health literacy [7] and one of its derivatives, namely physical literacy [8]. Physical literacy is one of the main goals of every health education program. SHAPE America [9] uses the following definitions when referring to physical literacy and health literacy. Firstly, physical literacy is defined as an individual’s ability, confidence, and desire to be physically active for life. Health literacy, on the other hand, refers to one’s capacity to make sound health decisions that lead to the adoption of healthy lifestyles now and later in life. Understanding the effectiveness of physical literacy in the context of health supports clinical, population, and school health programming. In fact, it is now widely recognized that, according to neuroscience, physical activity produces beneficial effects on our psycho-physical health [10].

In this context, physical activity in a school environment provides unique and extraordinary opportunities, in which one can work on the regulation of emotions while also favoring relationships with peers and the classroom climate [11]. In this way, emotional control and self-esteem are enhanced, and socialization and autonomy skills are increased, thus increasing the awareness of one’s potential and limits [12]. Schools, by nature being arenas of culture and science, can first and foremost become the place for culture, prevention, health and physical literacy, and health promotion—also in intergenerational terms (aimed not only at students but also at teachers and parents). The COVID pandemic has taught us how important it is to nurture our health and well-being. The school environment can become a place of education and discussion, starting from a necessary health and physical literacy standpoint, not only in the field of scientific subjects but also in the new transversal programming of all other disciplines present in schools’ curricula [13].

Therefore, this paper covers the themes of Italian schools’ medical services and is aimed at both capturing the current state of the art and reflecting on how physical activity—specifically active breaks—may promote psycho-physiological well-being among students.

## 2. Search Strategy, Identification of Studies, and Study Characteristics

This literature review offers a comprehensive examination of research studies that have covered the themes of Italian schools’ medical services aimed at both capturing the current state of the art and reflecting on how physical activity—specifically active breaks—may promote psycho-physiological well-being among students.

Four databases, namely Google Scholar, PubMed, Web of Science, and Cochrane Library, were employed to verify the collected studies. The studies were identified by implementing the subsequent Boolean search syntax: “((School medical service” or “medicine at school”) and (“active breaks” or “classroom-based physical activity”)”/“(“physical activity at school”) and (“psychological and physiological well-being” or “psycho-physiological well-being”))”. Later, a variety of filters were utilized: text readiness—full text; species—humans; languages—English; period—last 30 years. Lastly, additional screening facilitated the utilization of inclusion and exclusion parameters:

(i) English-language publications;
(ii) Time interval of studies between 1990 and 2023;
(iii) Physical activity or exercise as a tool for the improvement of cognitive function;
(iv) Study design: randomized controlled trials.

Studies were excluded from consideration if they failed to meet the specified inclusion criteria or if they did not sufficiently emphasize school medical services and/or school-based active breaks.

## 3. School Medical Service and Health Promotion: A Renewed Concept of Corporeality

Talking about health at school and the relationship between social and health services and schools means innovating approaches, strategies, and organizational models according to declinations that adapt to the cultural context and territorial characteristics of the country. At school, health promotion can, in fact, generate benefits and positive effects not only for students but also for families and communities (for example, thanks to the transmission of knowledge from the school environment to the family environment) [14]. By training and raising awareness among students, bringing social and health services closer to schools, and ensuring a healthy environment in the school context, it is possible to influence the determinants of health and ensure greater equity in the provision of social and health services, thereby facilitating access to them. In addition, the presence or connection of the school structure with health services could be a valuable tool for addressing frailties and managing any health-emergency situations of the students, especially those suffering from chronic diseases [15]. This approach requires, in particular, the structuring of paths for the acquisition of correct lifestyles that are effective both in the fight against childhood obesity and in the improvement of the psycho-physical factors of pupils [16]. In proposing a new model, it would be useful to draw on the opportunities offered by movement in renewed synergy between the body and mind. In this sense, physical activity can represent a more attractive and holistic way to incorporate the promotion of health and healthy lifestyles in the school context where context, psychosocial circumstances, individual abilities, and knowledge are considered [17]. Connecting multiple contexts for the promotion of physical activity increases the feasibility and likelihood of changing behavior and finding effective strategies to respond to the emergence of physical inactivity [18].

The body and movement, especially in the period of primary school, represent the mediators between the environment and the child. They allow for collecting and processing information and developing an adaptive response [19]. They are also a powerful means of expression and communication. Through the body and movement, children explore, learn, and develop adaptive functions, such as those that allow individuals to modify the external environment. In this way, children develop their cognitive and social skills, as well as motor skills [20]. Recent research based on neuroscientific theories shows that experiences through body and movement play an essential role in the development of the mind, and individual differences play an important role in the relationship between bodily experience and the mind [21]. Perception and the possibility of acting on perceptions determine knowledge. For these reasons, the psychological and pedagogical aspect of the body and its movement in the development of learning and social skills in early childhood education is a topic of interest in several studies. Research has shown that effective learning strategies can strengthen the mechanisms for modifying attitudes, knowledge, and behaviors related to lifestyle in early childhood [22].

School, particularly primary school, should play a central role in encouraging the practice of a wide variety of motor experiences. It should provide not only tools and opportunities for practice but also elements of knowledge and skills, which can guide choice, motivate participation, and last but not least, contribute to the well-being of pupils and teachers [23]. Schools represent fundamental places for the growth and education of people, processes that cannot fail to address the body, corporeality, and the numerous opportunities offered by the experience of movement. Therefore, it is essential to stimulate the expressive and communicative potential of the body in teaching.

## 4. Physical Activity and Implementation of Physically Active Lifestyles

The scientific literature attributes a decisive role of the regular practice of physical activity in the prevention of chronic non-communicable diseases and the improvement of psychophysical well-being and, thus, the quality of life of individuals in all age groups and of the community [24]. Contrarily, sedentary behaviors are one of the main health-risk factors; more than 800,000 deaths per year globally are attributable to physical inactivity.

Physical inactivity is a widespread problem in EU countries. According to data from the latest Eurobarometer survey, 45% of the people surveyed said they never exercise or engage in sport, and one in three people do not achieve sufficient levels of physical activity [25]. Finns are most likely to exercise or play sports at least once a week (71% of respondents), followed by people from Luxembourg (63%), The Netherlands (60%), and Denmark and Sweden (59%). In contrast, more than half of the respondents in eight countries said that they do not play sports, with the highest levels in Portugal (73%), Greece (68%), and Poland (65%). Today, Germany, Italy, and France contribute the highest burden of insufficient physical activity to health spending in the EU [26]. The result is that, in absolute terms, Italy is in second place for public health expenditure due to sedentary lifestyles; while Germany spends EUR 2.062 million, Italy exceeds France’s EUR 1.094 million with EUR 1.327 million. When measured per capita, Italy’s spending is still above the European average and after that of Malta, Belgium, Sweden, Germany, Ireland, and Portugal. Europe, as we know, is a complex continent, which offers a rich and varied panorama with respect to many aspects of our lives. This includes sedentariness, and territorial analysis of the phenomenon presents important discrepancies [27]. This calls into question the influence of the local culture. In some countries, more attention is paid to personal care and discerning between thoughtful food consumption and food abuse. Others are more permissive of the adoption of low-calorie diets and indolent to physical movement and sporting activity in general. Another variable that has great influence on the propensity to “play sports” appears to be the level of education. Aptitude for sport is directly proportional to this; a high level of education corresponds to a lower level of sedentariness. This is a trend that, while maintaining a ratio of gender differences, is particularly interesting for the analysis of the phenomenon, precisely because a sedentary lifestyle increases linearly as the level of education decreases [28]. Time is a fundamental variable in the choice of whether to practice physical and sporting activities. In the case of women, the profile of the typical sportswoman outlines a young, educated, employed, and childless woman. With respect to this last aspect, beyond the influence of their level of education, it is the absence of children for women that generates the possibility of devoting themselves to physical movement for their own well-being or pleasure. This is because, in some societies, there is still a rigid and sexist division of gender roles that culturally supports the stereotype that family care is considered a natural feminine task. Time is the most recurrent variable, which transversally explains, or justifies, sedentary behaviors. Even without drawing on the scientific literature or the results of surveys on the subject, there is a common feeling that the basis for a sedentary lifestyle is the lack of time due to work or study commitments. This, in fact, is the main reason that also recurs in the literature; i.e., the one most often stated by interviewees [29].

In this context, health promotion, which supports the adoption of healthy lifestyles and the fight against harmful ones, assumes a fundamental role that should not be carried out in only the health sector but in all areas of civil society and politics as a whole [30]. It follows that a multi-setting, multi-stakeholder, multi-component approach is needed to promote health. The variables involved in health promotion interventions and, therefore, also in the practice of regular physical activity are complex and varied. Over the years, the World Health Organization (WHO) has published a series of policy documents with the aim of providing countries with reference criteria for feasible and effective national policies and strategies. Focused exclusively on the promotion of physical activity, the “Global action plan on physical activity 2018–2030: More active people for a healthier world” [31] underlines how the fight against physical inactivity must pass through comprehensive and intersectoral actions, which intervene both in individual behaviors and the social, economic, and environmental determinants of lifestyles. In fact, the beneficial effects of physical activity can contribute directly and indirectly to the achievement of some of the Sustainable Development Goals of the UN 2030 Agenda [32]. These include reducing the use of fossil fuels and air pollution, reducing traffic, improving road safety, the sustainable development of cities, reducing premature mortality caused by chronic non-communicable diseases and health inequalities, and increasing gender equality. Socio-economic and environmental changes due to globalization, as well as increasing urbanization, have led to a significant increase in unhealthy lifestyles, including physical inactivity and sedentary lifestyles, and consequently, in the incidence of chronic non-communicable diseases.

## 5. Strategies Developed for Schools

The strategies developed for schools are in line with the objectives and actions promoted at the national and international levels. Different programs aim to prevent chronic non-communicable diseases and promote health through cross-sectoral policies and actions, according to an integrated and comprehensive approach [33]. School is an ideal setting to increase physical activity levels, as it is the place where children spend most of the day during childhood [34]. Numerous educational interventions have been proposed to encourage the practice of physical activity in a school context, most of which are structured as multi-component projects [35]. They are aimed at promoting active lifestyles and analyzing the relationships between increased levels of physical activity and its effects on cognitive functions, school performance, and related psychological–relational factors. The main actions and organizational methods that promote the practice of motor activities at the developmental age are approaches that fall into one of the following three categories [36]: (i) the expansion of opportunities for children to be active (e.g., doing activities before they enter school, at the end of classes, or during recess); (ii) the extension of existing opportunities to practice physical activities (e.g., increasing the amount of time/sessions related to physical education, increasing the number of hours per week of physical education, or introducing afternoon sports); (iii) enhancement of opportunities to carry out the motor activities already present in the curriculum through strategies aimed at increasing the time of motor engagement; i.e., lessons in which the students are physically active (e.g., increased availability of equipment, increase in options related to the various sports activities that can be practiced, and identification of spaces and environments to be used at the same time).

In schools, physical activity can be promoted during other lesson hours, recess, and breaks. By choosing to alternate and integrate classical and traditionally theoretical teaching with enactive and integrated teaching strategies, some topics in disciplines such as mathematics, geography, and foreign languages can be taught starting with experiential situations followed by theoretical moments [37]. In this sense, one promising approach is represented by active breaks (ABs), which have been identified as a crucial strategy to enhance children’s psycho-physical skills in their early years of education. Additionally, it has been found that ABs significantly improve early childhood social skills, allowing children to enjoy learning according to their learning styles [38]. They consist of carrying out physical activity during curricular hours, alternating many sedentary moments with short-term physical activities that can also benefit other school skills. According to a recent review conducted by Watson, Timperio, Brown, and Heskethet [39], the modalities for Abs that are currently proposed, in relation to timing, objectives, and organizational methods, are as follows:

1. Active breaks between two successive lessons;
2. Active breaks within a lesson;
3. Physically active lessons, with the integration of physical activity into other disciplinary courses (e.g., geography, mathematics, geometry, history, etc.).

**Figure 1:**
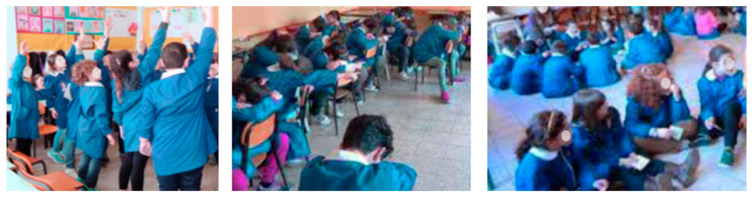
Classroom-based physical activity.

Several studies have shown that, in fact, short active breaks are effective at increasing physical activity levels, enjoyment, and the desire to learn [40,41,42]. However, the requests and directives from teachers, a very large and varied school curriculum, greater attention given to standardized tests, and some constraints related to educational institutions are among the main factors that limit the planning of such interventions in schools [43]. For this reason, periods of active breaks are considered important from an educational point of view and have great pedagogical value because they provide the opportunity to acquire various skills that are useful for the child’s life: the ability to collaborate, manage relationships among peers, follow common rules, confrontation, and autonomy [44]. In summary, we can describe ABs as small intervals characterized by the practice of physical activity carried out by teachers during daily disciplinary activities within the classroom context during curricular lessons [45]. They were started with the intention of increasing the amount of daily physical activity in the current rather sedentary childhood generations. Over time, they have proven to be effective at improving the psycho-physical well-being of children, their behavior, and the overall climate of the class, as well as in teachers. In fact, their practice seems to improve the quality of school life, including social behavior, attention, concentration, and involvement in activities [46]. ABs offer teachers the possibility of organizing breaks in relation to different factors and situations, making them very flexible and adaptable to the school context and specific classes [47]. Their duration can range from 3 to 15/20 min, and their realization, generally in the classroom near one’s desk, has also opened to the outdoor environment, such as the school playground, where some teachers feel safer and better able to conduct the planned activities [Figure 1].

The aim of this contribution is to provide a framework for the effects of ABs in primary school on: (i) the levels of physical activity and improvement in overweight/obesity; (ii) academic performance; (iii) related psychological factors, highlighting their strengths and weaknesses regarding their rational proposal.

## 6. Effects of Active Breaks and Physically Active Classes

### 6.1. Physical Activity Levels and Reduction of Overweight/Obesity Status

The perception of obesity as a priority public health problem, and consequently, the need to carefully monitor the nutritional situation of the general population, and especially that of children, is one of the main objectives of school medical services [48]. The most recent studies in the field warn that the acquisition of unhealthy lifestyles and the phenomenon of physical inactivity continue to progress, with percentages of overweight adolescents having more than tripled in the last 20 years [49]. According to the available data, unfortunately, young people today spend most of their free time in sedentary activities, mainly watching television and playing computer games [50]. A sedentary lifestyle and lack of physical activity are unhealthy behaviors that are harmful from childhood. They continue into adolescence and are often accompanied by other risky behaviors, which, in adulthood, can cause health problems and chronic diseases [51]. There is clearly a need for greater emphasis on improving quantitative and qualitative opportunities for physical activity, especially since the prevalence of obesity appears to be more related to lack of movement than just increased food intake [52]. In this sense, prevention, far from the traditional negative connotation of the biomedical model, is aimed at increasing the capital of the first years of life, addressing those who are most at risk, and protecting children from involvement in risky behaviors. Promotion and prevention are, therefore, part of the concept of salutogenesis, which literally means the search for the sources of health. From a salutogenic perspective, health promotion and prevention pursue empowerment; i.e., “making individuals, families, and communities able to take control over their lives and their environment, that is, to acquire an active role towards their environment and their existence” [53]. The benefits of physical activity for health, especially for overweight and obesity, have long been documented. Despite the fact that the WHO guidelines recommend at least 1 h a day of physical activity among children aged 5 to 17, about 80% of children and adolescents remain physically inactive [54].

School is the ideal environment for carrying out surveillance for reasons of operational efficiency, as children are concentrated there at the same time, and for reasons of usefulness in view of the necessary interventions. Therefore, in the 6–14 age group, it is a priority to promote physical activity and counteract the widespread tendency toward a sedentary lifestyle. School is an ideal setting for countless reasons. It is the place where children spend a lot of time from early childhood; it is inclusive and overcomes numerous barriers (resources, availability, costs), which are often the main obstacles to the practice of extracurricular physical activities; and it offers multiple opportunities during the days and weeks spent at school, starting with physical education lessons [55].

ABs represent an evidence-based intervention to promote physical activity and reduce the sedentary lifestyle of pupils during the school day, as they are organized in most Italian schools, starting from primary school. ABs are recommended by the American Community Guide.

Two systematic reviews [56,57], both addressing primary school students regarding breaks and active lessons, reported the following results related to the time of intervention or school day:

- Increased time for moderate to vigorous physical activity during school;
- Increase in the number of steps during the school day;
- Acquisition of behaviors oriented toward healthy and correct lifestyles.

The study by Bobe, Perera, Frey, and Frey [58] highlights not only how these methods are effective at increasing physical activity levels in a sample of primary school children but also how such strategies are favorably welcomed and used by the teachers themselves, generating continuity of the interventions. Moreover, Simon et al., in a study published in 2008 [59], provided evidence that a multilevel intervention program aimed at promoting an active lifestyle among pupils led to a notable increase in their physical activity (approximately 1 h per week), also resulting in a decrease in their time spent watching TV and videos. Additionally, the findings of their study demonstrated that the intervention program effectively prevented excessive weight gain in non-overweight adolescents, as evidenced by a 50% reduction in the incidence of overweight over the course of 4 years. In a similar way, Li and colleagues [60], in a non-randomized controlled trial, showed that the implementation of active breaks was effective at reducing body mass index (BMI), skinfold thickness, and fasting glucose levels while concurrently enhancing the duration of moderate-to-vigorous physical activity (MVPA). Their study demonstrated that the alteration in BMI within the intervention group (−0.02 ± 0.06 kg/m^2^) had a statistically significant disparity when compared with the control group’s BMI change (0.41 ± 0.08 kg/m^2^). The adjusted average variance was determined to be −0.43 kg/m^2^ (95% CI: −0.63 to −0.23 kg/m^2^, *p* < 0.001). Such results inherently substantiate the necessity for the formulation of successful and practicable interventions aimed at combatting obesity within school environments.

### 6.2. Cognitive Enhancement

In a broader framework of analysis related to the connection between school medicine and physical activity, we investigated how movement and how much of it can affect cognitive and learning development in this context. In fact, limiting the meaning of physical activity to the sole objective of good health seems rather reductive. So much so that a large body of literature shows it has beneficial effects on cognitive functions, attention, concentration, memory [61,62,63,64,65] and, indirectly, on school results [66,67,68], as well as on the general well-being of children and, consequently, teachers [69]. In fact, the knowledge base derived from research data provided by neuroscience suggests that physical activity has the potential to encourage the brain to function at its optimal level of capacity, thus promoting the multiplication of neurons and the strengthening of neuronal connections. The most recent research results have allowed a better understanding of the role that physical activity can have on brain structure and related neurobiological changes [70], focusing on the influence that it can exert on neuronal plasticity. The concept of plasticity is fundamental to understanding how physical activity can optimize brain function by promoting the quality of learning. Neuroplasticity is a constant and continuous process that is capable of modifying existing neuronal networks by mediating the structural and functional adaptations of synapses in response to changes in behavior [71]. Physical activity, especially ABs, can stimulate the production of brain neurotrophic factor (BDNF), which encourages the growth of new brain cells. It is the key biological link between physical activity, learning, and academic success. In addition, high levels of physical activity seem to result in brain changes due to the increased oxygenation and irrigation of tissues, as well as increased metabolic activity, promoting improved neurodevelopment. It is hypothesized that carrying out a normal physical activity routine leads to relevant changes in neurogenesis and angiogenesis and improves central nervous system metabolism [72]. The process of neurogenesis takes place mainly in the hippocampus, an area specialized in spatial learning and the consolidation of short- and long-term memory. Although some studies have reported small negative associations, most scientific research has now amply demonstrated how the effects of physical activity on the brain can lead to positive outcomes on school performance.

Using movement in the classroom as a method to teach is a valuable strategy to enhance the learning process and provide all students with the opportunity to grow cognitively, socially, and physically. Its purposes are to: (i) prepare the brain; (ii) provide brain breaks; (iii) develop class cohesion; (iv) review content; and (v) teach content. In fact, several studies showed that short periods of active breaks are effective at increasing the levels of attention, concentration, and enjoyment and reducing inappropriate behavior in the classroom. They are also able to put students in the optimal condition to learn, improving their academic performance. Comparing the improvement in standardized test scores, children who attended active classes performed 6% better than their peers who received the same classes inactively. Donnelly et al. [73], in a review of the literature, argued that the regular and constant practice of physical activity, combined with short breaks introduced during curricular lessons aimed at increasing the opportunities to carry out motor practice, positively stimulated the cognitive functioning of children. Some studies also showed the existence of a positive correlation between the performance of physical activity carried out before a curricular lesson and the subsequent behavior held in the classroom by children. Their attention, concentration, and memory were especially improved.

In addition, from the analysis of the results of several studies evaluating the duration of school physical activity, it was concluded that individual physical education lessons can improve a student’s attention but have no effect on the stimulation of executive functions or school performance. On the contrary, the integration of physical activity within other curricular subjects makes it possible to better stimulate executive functions and academic performance compared with the isolated lesson. This is especially the case if the intervention programs involve aerobic activities involving cognitive difficulties [74]. Therefore, the introduction of ABs during the school day is an advantageous strategy to improve behavior during subsequent curricular activities in order to better engage the students, allowing them to be focused on learning goals and creating a motivational climate oriented toward competence.

Blom Skrade [75] analyzed the relationship between the number of hours of physical education and the academic performance of adolescents, integrating a physical activity program known as Move for Thought into the school mathematics curriculum (M4T). The study found that the integration of physical activity during other curricular lessons in the classroom improved the learning of mathematics. The findings revealed that school-based physical activity intervention had a beneficial impact on the students’ abilities in mathematics, as demonstrated by a 45% success rate. The author concluded that this program allowed the children to optimize their motivation in relation to achieving academic success; this perceived competence had a positive impact on mathematical performance, providing a unique and positive contribution to the subject. Similarly, Resaland et al. [76] investigated the effects of a 90 min physical education program, followed by a 5 min active break during recess at school and 10 min of physical activity at home. The subgroup analyses conducted by the researchers uncovered a beneficial impact of the intervention for individuals who exhibited the lowest levels of numeracy at the beginning of the study (i.e., in the lowest tertile). This effect was found to be statistically significant (*p* = 0.005) in the context of the subgroup ∗ group interaction. In comparison with the control group, the intervention group demonstrated a standardized difference of 0.62 (95% CI 0.19–1.07). The results of the study led the authors to hypothesize the existence of a greater stimulation of learning.

Therefore, adherence to structured physical activity programs during the school day, together with different opportunities to have an active life in free time, should be the most important vehicle for promoting the physical and cognitive health of children. Thus, it is up to schools to make physical education the focus of the school day in order to facilitate learning for all students and provide motivation to enable them to achieve greater academic success.

### 6.3. Development of Psycho-Affective and Social Factors

Several studies showed that those who led a more active lifestyle exhibited different development of psycho-affective factors, greater problem-solving skills, more concrete emotional stability, greater effectiveness in creativity, and higher levels of attention and concentration. The effects on improvements in behavioral, cognitive, and psychological factors are consistent with the international literature analyzed. A recent review of the literature argued that regular practices of physical activity, short breaks, and educational interventions aimed at increasing opportunities for motor practice positively stimulated children’s cognitive functioning [73]. Some studies showed a positive association between the practice of physical activity before class (e.g., during break/recess) and behavior in the classroom during subsequent lessons [77]. One study reported a slight to moderate improvement in focused attention following the proposal of active breaks for primary school students [41]. Short-term educational interventions also provide experiences involving the whole class. They stimulate fun, intrinsic motivation, and personal success, regenerate and expand relationships among peers, and help children to develop social skills otherwise not acquired in the more structured school environment. Thus, the classroom environment and other spaces of the school become pleasant, emotionally engaging places, in which the usual curricular school day, based on theoretical teachings, is interrupted by bodily-motor experiences that give meaning and direction to all learning [78]. Physical activity, in fact, does not only imply goals related to motor skills but goes beyond that. The body in motion promotes self-awareness, making people experience the potential and limits of their physicality for the development of self-control toward themselves and others. Physical activity during lessons, playing in the open air, and the use of small tools encourage the adoption of good practices for nutrition [79], personal hygiene, and health education in general [80]. In addition, in the growth process, physical activity in the school environment invites students to reflect on changes in the body, as well as through comparison with their peer group. The result is experiences that involve cognitive, social, cultural, and affective aspects. The body becomes an increasingly adequate and effective tool for communication and relationships. Experiencing victory or defeat promotes the control of one’s emotions, capacity for resilience, and self-evaluation. Physical activity and teamwork often encourage the expression of discomfort and messages that verbal communication struggles to decode. In this way, it becomes easier for teachers to identify behavioral or relationship problems and then intervene, personalizing the activity. Cooperation and teamwork are conveyed through physical activity, as well as self-respect and respect for the opponent, loyalty, a sense of belonging, and responsibility [81]. This approach makes it possible to move from teaching based on notional learning to an approach that places the active involvement of the pupils at the center. Physical activity in the classroom allows students to learn strategies such as participation, collaboration, autonomy, and responsibility in group moments. Group learning is fundamental in the acquisition of skills, as it is in groups that many social interactions occur. It can be used to create situations in which members can learn, share, and experience, which is the basic process of any dynamic that develops and takes place in team games.

Physical activity in the classroom makes it possible to offer moments of well-being and motor recovery and to achieve personal autonomy and gratification, proving it to be a concrete educational tool. The promotion and activation of re-elaborating physical activity takes on particular significance for people with disabilities who increasingly feel the need to be able to also express themselves through the body and movement [82]. These activities, implemented within the school environment, allow disabled students to escape the recovery and socio-educational contexts in which re-education interventions and sports activities dedicated only to the disabled are mainly carried out. An aspect that turns out to be fundamental is the drive to act derived from the motor dimension. Physical activity in the classroom for disabled pupils provides an opportunity to give space to their abilities, even if reduced, and what they are able to do in relation to the world in which they live, and in which, very often, only what is missing and what they are unable to do is considered [83]. The greater autonomy of a pupil with disabilities in terms of action has repercussions for learning to the point of involving the affective sphere: the effectiveness of actions is favored, allowing for the structuring of self-esteem. Physical activity, in addition to promoting the consolidation of cognitive functions, thus becomes a reason for emancipation and growth. Giving space to spontaneous motor skills and working for motor education is essential for the full development of children with disabilities and acts as a permanent support for the realization and participation in “active” proposals during their growth span.

In the investigation conducted by Schmidt et al. [84], the authors presented evidence that alterations in the positive emotional state throughout the interventions acted as a mediator between cognitive involvement and concentrated attention, as well as between cognitive involvement and processing speed. Significant effects among the groups were observed, favoring the intervention group in relation to the variable of self-esteem (which encompasses general, academic, and social self-concept/self-esteem), as well as body image (including physical self-concept), with a specific intervention. Similarly, Christiansen et al. [85] conducted an analysis to identify disparities between an intervention group and a control group. These differences were adjusted for variables such as gender, age group, social class, recreational sport, and body image. In the study, the control group experienced an increase in physical self-concept, but only among students who perceived themselves as having a thinner body image. Conversely, the intervention group exhibited an increase in physical self-concept among all students. Moreover, it was observed that the intervention group students who did not engage in recreational sports demonstrated greater improvement in their general self-esteem compared with their counterparts in the control group.

Physical activity also plays an important role in the process of social integration, as it allows teachers and families to create bonds and think about life pursuits outside of school for disabled children [86]. Physical activity in the classroom or active breaks are tools for personal and collective growth. If the teacher succeeds in making all students actively participate through the creation of a climate of relationships that stimulates the well-being of the children, disability will not be perceived as a limit either by disabled children or their classmates. Furthermore, it will not be considered an obstacle to carrying out the teaching activity.

## 7. Active Breaks in the Italian Experience

The Italian school system is full of projects aimed at promoting physical activity, especially among primary school children. However, the actions implemented so far are fragmented, fail to involve all the target pupils, and often have problems of continuity over time [87]. In fact, in the Italian school system, this intervention strategy is not given the right importance. The general trend in Italy is far from what is good pedagogical practice. The teaching of physical education in schools is given less and less importance than other curricular subjects considered essential for the cognitive growth of pupils [88]. This aspect is worrying, especially when related to what has been stated in recent years by cognitive neuroscience. They argue that most cognitive processes are grounded in the human body’s perceptual and physical interactions with the world [89]. Until now, in fact, the cultural debate on the importance of physical education for the psycho-physical well-being of children has developed mainly in specialist and scientific fields, increasingly often outside the school framework. A comparison of different European school systems shows that the Italian school system still has numerous critical points at both methodological and didactic levels and political and organizational levels [90]. This reflects a system that has invested little in terms of quality and quantity. This situation has generated a lack of motivation on the part of the teachers, in whom little training has been invested, and a widespread lack throughout the country of spaces specifically dedicated to physical activity. In order to systematize the activities and make them usable by schools throughout the country, it is necessary to select evidence-based interventions that have put in place good practices that have been consolidated for some time. A training action is needed to transfer knowledge and skills, promoting the empowerment of the school so that it becomes the main protagonist in terms of promoting a physically active lifestyle [91].

In Italy, active breaks were promoted and became particularly popular after their inclusion in the “Guidelines on Physical Activity” (Ministry of Health, 2019). These strategies imply a multi-component approach; i.e., the use of interventions aimed at improving the quality of curricular physical education through collaboration with external partners for the implementation of complementary measures (e.g., active transport, education to correct eating habits). The aim is to promote interdisciplinary and transversal strategies for the promotion of motor activities [92]. Multi-component projects, in fact, allow schools to take advantage of all opportunities for pupils to be physically active throughout the school day.

Among the interventions aimed at primary schools, Table 1 presents some examples of good practice carried out on Italian national territory.

## 8. Active Breaks Projects around the World

On the international scene, there are several projects, especially conducted in primary schools, aimed at preventing sedentary habits, promoting healthy lifestyles, and increasing the levels of physical activity in children [93].

An example of good practice in Europe is the multi-component project "Active Living" [94]. To increase the physical activity levels and reduce the sedentary behaviors of primary school children, the project, conducted by recruiting 1343 pupils from 21 primary schools in the Netherlands, integrated three different active break interventions: (i) at school, (ii) on the way to school, and (iii) during leisure time. In Belgium, a significant project has been developed to promote physically active lifestyles among primary school children. Based on the San Diego State University SPARK program [95], it included: (i) a school physical education program, (ii) classroom nutrition education classes, and (iii) an extracurricular physical activity program. The original SPARK program has been adapted to the Belgian educational and cultural system, promoting physical activity even during rest periods and lunch breaks, as primary schools in Belgium have several rest periods distributed equally throughout the school day and longer lunch breaks than US primary schools.

In the USA, multi-component projects, such as “SPARK”, “CATCH”, and “Go for Health” are now a well-established reality in the promotion of active and healthy lifestyles in children. The SPARK (“Sports, Play, and Active Recreation for Kids”) project integrates a physical education program carried out at school with an extracurricular physical activity program self-managed by the children themselves. Along the same lines, the *CATCH* (Child and Adolescent Trial for Cardiovascular Health) [96] and Go for Health [97] projects were designed to increase children’s involvement in moderate to vigorous intensity motor activities (MVPA) and to promote healthier behaviors oriented toward active lifestyles to be maintained throughout the course of life.

The “Pathways” project includes four areas of intervention: (i) theoretical lessons regarding correct habits and lifestyles, (ii) dietary intervention, (iii) active breaks in the classroom, and (iv) family involvement. The project lasted 6 years, during which 41 schools from 7 communities of American Indians participated in the intervention. All schools worked in collaboration with an academic institution partner in the project (White Mountain Apache and San Carlos Apache—Johns Hopkins University, Baltimore; Navajo—University of New Mexico, Albuquerque; Sicangu Lakota and Oglala Lakota—University of Minnesota, Minneapolis; Tohono O’odham Nation and the Gila River Indian Community—University of Arizona, Tucson). The University of North Carolina served as a coordinating center [98].

## 9. Discussion

The results of this review underline the importance of school medicine in carrying out disease prevention and health promotion activities for pupils in compulsory education. The review also proposes interventions and strategies that are congruent with public health priorities and supported by adequate scientific evidence. Among these, physical activity interventions, and in particular ABs, represent an optimal strategy to intervene in the direct link between health, educational achievement, and quality of life.

Given the multi-component nature of health-risk factors, it is necessary to adopt an approach that also considers causes external to the health sector. This is possible by involving those sectors, such as schools, that can play a crucial role in the dissemination and maintenance of healthy lifestyles but, at the same time, also provide skills and resources in favor of increasing the levels of physical activity. In this context, the international and national strategies for the promotion of physical activity of the last decade, characterized by an integrated, intersectoral, and partnership-oriented perspective, are applied [99].

Based on this assumption, the approach proposed in the school environment as an intervention to acquire physically active lifestyles is systemic and provides coordination between schools and school medicine services. The Italian strategy by which the policies to promote health are carried out, therefore, is in accordance with the international framework. This strategy aims to avoid piecemeal interventions by taking into account all determinants acting on health-related lifestyles that impact individual and collective well-being. From this perspective, the entire school community, in concert with the school medicine service, must work in synergy to plan and implement structured and coordinated interventions to promote physical activity and to spread the culture of an active lifestyle as a fundamental tool for well-being. By identifying some predefined programs, they must aim to systematize validated and consolidated methods, tools, and good practices [100].

In this context, active breaks can represent an effective response that is sustainable over time with respect to two fundamental needs: (a) as a public health strategy aimed at increasing the daily amount of physical activity and reducing the time spent doing sedentary activities; and (b) as a strategy for an innovative school capable of eliciting a sense of psycho-physiological well-being during the school day, for both pupils and teachers, and improving behavior, attention, and participation in the various curricular activities proposed [101].

With this strategy, the classroom, in a holistic sense, is transformed into an environment in which not only are aspects related to disciplinary knowledge and skills addressed, but it also intervenes in broader and more decisive aspects of the very life of the person who, in reality, is at the center of their formation process, with real needs and requirements. It goes without saying that these activities are not a substitute for physical education. They act as sensorimotor reminders that facilitate participation in school life by reactivating the students, too often anesthetized at their desk, sitting on their chairs in passive and incorrect bodily postures. Beyond the direct effect on health, which certainly cannot be completely resolved with active breaks, the most striking aspects of this approach lie in the mental availability for physical activity, in general, and in the improvement of behavior in the classroom owing to the energizing effect attributable to them, especially for children unable to maintain attention [102]. Some studies showed that cognitively and academically engaging active breaks (i.e., made with reference to disciplinary knowledge studied in the curriculum (mathematics, languages, science)) produce the best effects on children’s cognitive functions and learning [103].

Active breaks, as well as other proposals for sensory–motor activities, must be part of children’s curricular experience and integrated into the pedagogical framework that characterizes teaching–learning activities, becoming decisive and effective components for the optimal development of each person. It is not, therefore, a question of finding additional space and time but of characterizing a daily school life that considers all the educational complexity of each individual, and not only that referable to individual disciplinary teachings. In this educational complexity, ABs must find an appropriate place as an integration into teaching strategies aimed at improving the learning environment and motivation toward training paths.

## 10. Conclusions

The school setting is one of the main areas of life during the developmental age. It is fundamental to implement actions that promote psycho-physical well-being, learning, social relationships, and the acquisition of correct lifestyles through a global and systemic approach that allows health to be promoted in its broadest sense. Schools that promote health emerge and develop from a strong collaboration between the school system and the health system, with the support of families. Health promotion at school encompasses both the health education taught in the classroom and all the efforts dedicated to creating a context, school policies, and a curriculum aimed at making healthy options more accessible. A school that promotes health is much more than a school that carries out health-promotion activities. It is a school that takes a global approach to health promotion. It is identified as the institution par excellence that can play a decisive role in promoting well-being, lifestyles, and healthy behaviors in the youth population. Children spend a large part of their lives at school and, if properly guided, they can acquire the knowledge, skills, and competencies to choose healthy lifestyles. A school that orients not only the curricula but the organization of the entire school community toward the promotion of healthy and active lifestyles represents a favorable context for students to develop the knowledge, skills, and habits necessary to live in a healthy and active way, even in adulthood.

In this context, the fundamental objective is to increase the number of people who, by choosing an active and healthy lifestyle through the joy of moving and the practice of sport, develop their potential in all domains (motor, cognitive, creative, affective, and social) from the transversal perspective of active and participatory citizenship.

## Figures and Tables

**Table 1:** Multi-component classroom-based physical activity projects in Italian schools.

Project	Areas of Intervention	Partners	Purpose
SBAM! Salute, Benessere Alimentazione, Movimento a scuola	Nutrition education; development of motor skills; active transport (pedibus).	Apulia Region Departments of Health (Food), Sport and Mobility (Pedibus), with collaboration of the following partners: University of Foggia, Laboratory of Didactics of Motor Activities; Regional School Office of Puglia; CONI–Puglia Regional Committee; CIP–Regional Paralympic Committee.	To educate to correct eating habits, to teach motor skills during curricular physical education lessons, and to promote active transport by pedibus.
Più sport @ scuola	Development of motor skills and knowledge of the different sports disciplines at school and extracurricular motor activities.	Veneto Region in collaboration with the Regional School Office for Veneto, University of Verona, and University of Padua.	To promote and increase the practice of recreational motor skills for the psycho-physical development of young people and the adoption of more active lifestyles.
A scuola di gioco sport	Introduction of different sports disciplines (in the form of games) within individual classes.	Municipality of Ascoli Piceno; Ministry of Education; School Office of the Province of Ascoli Piceno; C.O.N.I. Provinciale Ascoli Piceno.	To promote physical and sports education for all children, including the disabled, through a multi-sport and multilateral recreational sports methodology, and to develop the knowledge and practice of as many sports activities as possible.
Samba	Nutrition education; development of motor skills; active transport (pedibus and dog-walking); involvement of families.	University of Bologna, Department of Medicine and Public Health, Faculty of Exercise Sciences; Department of Educational Sciences; Local Health Authority of Bologna, Department of Public Health; Provincial School Office; Uisp Bologna.	To improve children’s awareness of the actions to be taken to achieve suitable lifestyles and motivate them to experiment with new behaviors (food and motor) that are able to turn into habits, with positive effects also on the level of self-esteem and interpersonal respect.
Scuola in movimento	Nutrition education; development of motor skills.	Abruzzo Region; CONES; Abruzzo Regional School Office; Universities of Chieti-Pescara and L’Aquila	To promote the adoption of healthy eating habits and guide children toward an active lifestyle.
A scuola di sport–Lombardia in gioco	Development of motor skills; healthy lifestyles; school inclusion.	Lombardia Region; CONES; CIP; ANCI; Lombardy Regional School Office.	To promote and implement physical activity and healthy lifestyles in primary schools, and to teach motor skills that contribute to the global development of the personality considered not only from the physical but also from the cognitive affective and social points of view.

## Data Availability

The data presented in this study was obtained from the included studies and was openly available.

## References

[1] World Health Organization (2003). Health and Development through Physical Activity and Sport.

[2] Keeton V., Soleimanpour S., Brindis C.D. (2012). School-based health centers in an era of health care reform: Building on history. Curr. Probl. Pediatr. Adolesc. Health Care.

[3] Veltro F., Ialenti V., Iannone C., Bonanni E., Morales García M.A. (2015). Promoting the psychological well-being of Italian youth: A pilot study of a high school mental health program. Health Promot. Pract..

[4] Bains R.M., Diallo A.F. (2016). Mental health services in school-based health centers: Systematic review. J. Sch. Nurs..

[5] Ricciardi W., Tarricone R. (2021). The evolution of the Italian national health service. Lancet.

[6] Ludici A., Tassinari Rogalin M., Turchi G. (2021). Health service and school: New interactions. Comparison between the Italian system and the Swedish system on the diagnostic process of pupils. Int. J. Incl. Educ..

[7] Paakkari L., Okan O. (2020). COVID-19: Health literacy is an underestimated problem. Lancet Public Health.

[8] Cornish K., Fox G., Fyfe T., Koopmans E., Pousette A., Pelletier C.A. (2020). Understanding physical literacy in the context of health: A rapid scoping review. BMC Public Health.

[9] Young L., O’Connor J., Alfrey L., Penney D. (2021). Assessing physical literacy in health and physical education. Curric. Stud. Health Phys. Educ..

[10] USA 1: SHAPE America Web Page—Physical Literacy. https://www.shapeamerica.org/events/physicalliteracy.aspx?hkey=61893e49-8a9e-430c-b4f5-8267480cb421.

[11] Shen B., McCaughtry N., Martin J. (2007). The influence of self-determination in physical education on leisure-time physical activity behavior. Res. Q. Exerc. Sport.

[12] Whitehead J.R., Corbin C.B., Fox K.R. (1997). Self-esteem in children and youth: The role of sport and physical education. The Physical Self: From Motivation to Well-Being.

[13] Braksiek M., Pahmeier I., Gröben B., Lindemann U. (2022). Implementation of physical activity-based health promotion measures in schools—Examples and evaluations from Germany. Sustainability.

[14] Ekwaru J.P., Ohinmaa A., Dabravolskaj J., Maximova K., Veugelers P.J. (2021). Cost-effectiveness and return on investment of school-based health promotion programmes for chronic disease prevention. Eur. J. Public Health.

[15] Massari E., Brgonzoli L., Crepaldi C., Iardino M., Fuschi D. (2023). New perspectives for the school medical service in Italy. Popul. Med..

[16] Rocha H.H.P., da Silva H.M. (2023). Schooling and Medical Assistance. The Curriculum of the Body and the School as Clinic: Histories of Public Health and Schooling.

[17] Leger L.S., Buijs G., Mohammadi N.K., Lee A. (2022). Health-Promoting Schools. Handbook of Settings-Based Health Promotion.

[18] Vaquero-Solís M., Tapia-Serrano M.A., Hortigüela-Alcalá D., Jacob-Sierra M., Sánchez-Miguel P.A. (2021). Health promotion through movement behaviors and its relationship with quality of life in spanish high school adolescents: A predictive study. Int. J. Environ. Res. Public Health.

[19] Andermo S., Hallgren M., Nguyen T.T.D., Jonsson S., Petersen S., Friberg M., Elinder L.S. (2020). School-related physical activity interventions and mental health among children: A systematic review and meta-analysis. Sports Med.-Open.

[20] Pascoe M., Bailey A.P., Craike M., Carter T., Patten R., Stepto N., Parker A. (2020). Physical activity and exercise in youth mental health promotion: A scoping review. BMJ Open Sport Exerc. Med..

[21] Schmidt M., Benzing V., Wallman-Jones A., Mavilidi M.F., Lubans D.R., Paas F. (2019). Embodied learning in the classroom: Effects on primary school children’s attention and foreign language vocabulary learning. Psychol. Sport Exerc..

[22] Cairney J., Dudley D., Kwan M., Bulten R., Kriellaars D. (2019). Physical literacy, physical activity and health: Toward an evidence-informed conceptual model. Sports Med..

[23] Bang H., Won D., Park S. (2020). School engagement, self-esteem, and depression of adolescents: The role of sport participation and volunteering activity and gender differences. Child. Youth Serv. Rev..

[24] Caldwell H.A., Di Cristofaro N.A., Cairney J., Bray S.R., MacDonald M.J., Timmons B.W. (2020). Physical literacy, physical activity, and health indicators in school-age children. Int. J. Environ. Res. Public Health.

[25] Santos A.C., Willumsen J., Meheus F., Ilbawi A., Bull F.C. (2023). The cost of inaction on physical inactivity to public health-care systems: A population-attributable fraction analysis. Lancet Glob. Health.

[26] Katzmarzyk P.T. (2023). Expanding our understanding of the global impact of physical inactivity. Lancet Glob. Health.

[27] Grandes G., García-Alvarez A., Ansorena M., Sánchez-Pinilla R.O., Torcal J., Arietaleanizbeaskoa M.S., Sánchez A. (2023). Any increment in physical activity reduces mortality risk of physically inactive patients: Prospective cohort study in primary care. Br. J. Gen. Pract..

[28] Ryom K., Simonsen C.B., Eshøj S., Nielsen G., Troelsen J., Maindal H.T. (2023). Tackling physical inactivity in Scandinavia: A narrative review of reviews supplemented by expert interviews. Scand. J. Public Health.

[29] Bueno-Antequera J., Munguía-Izquierdo D. (2023). Physical Inactivity, Sedentarism, and Low Fitness: A Worldwide Pandemic for Public Health. Integrated Science of Global Epidemics.

[30] Jakicic J.M., Kraus W.E., Powell K.E., Campbell W.W., Janz K.F., Troiano R.P. (2019). Physical Activity Guidelines Advisory Committee Association between bout duration of physical activity and health: Systematic review. Med. Sci. Sports Exerc..

[31] World Health Organization (2019). Global Action Plan on Physical Activity 2018–2030: More Active People for a Healthier World.

[32] UN Transforming Our World: The 2030 Agenda for Sustainable Development (UN, New York, 2015). http://bit.ly/TransformAgendaSDG-pdf.

[33] Deschesnes M., Martin C., Hill A.J. (2003). Comprehensive approaches to school health promotion: How to achieve broader implementation?. Health Promot. Int..

[34] Lavin A.T., Shapiro G.R., Weill K.S. (1992). Creating an agenda for school-based health promotion: A review of 25 selected reports. J. Sch. Health.

[35] Herlitz L., MacIntyre H., Osborn T., Bonell C. (2020). The sustainability of public health interventions in schools: A systematic review. Implement. Sci..

[36] Martín-Rodríguez A., Gostian-Ropotin L.A., Beltrán-Velasco A.I., Belando-Pedreño N., Simón J.A., López-Mora C., Navarro-Jiménez E., Tornero-Aguilera J.F., Clemente-Suárez V.J. (2024). Sporting Mind: The Interplay of Physical Activity and Psychological Health. Sports.

[37] Vazou S., Webster C.A., Stewart G., Candal P., Egan C.A., Pennell A., Russ L.B. (2020). A systematic review and qualitative synthesis resulting in a typology of elementary classroom movement integration interventions. Sports Med.-Open.

[38] Mullins N.M., Michaliszyn S.F., Kelly-Miller N., Groll L. (2019). Elementary school classroom physical activity breaks: Student, teacher, and facilitator perspectives. Adv. Physiol. Educ..

[39] Watson A.J.L., Timperio A., Brown H., Hesketh K.D. (2018). A pilot primary school active break program (ACTI-BREAK): Effects on academic and physical activity outcomes for students in Years 3 and 4. J. Sci. Med. Sport.

[40] Rasberry C.N., Lee S.M., Robin L., Laris B.A., Russell L.A., Coyle K.K., Nihiser A.J. (2011). The association between school-based physical activity, including physical education, and academic performance: A systematic review of the literature. Prev. Med..

[41] Donnelly J.E., Lambourne K. (2011). Classroom-based physical activity, cognition, and academic achievement. Prev. Med..

[42] Mahar M.T. (2011). Impact of short bouts of physical activity on attention-to-task in elementary school children. Prev. Med..

[43] Wadsworth D.D., Spring K.E. (2024). The Impact of an Acute Active Reading Intervention on Physical Activity Levels in Preschoolers: A Comparative Analysis. Children.

[44] Turner L., Chaloupka F.J. (2016). Reach and implementation of physical activity breaks and active lessons in elementary school classrooms. Health Educ. Behav..

[45] Muntaner-Mas A., Morales J.S., Martínez-de-Quel Ó., Lubans D.R., García-Hermoso A. (2024). Acute effect of physical activity on academic outcomes in school-aged youth: A systematic review and multivariate meta-analysis. Scand. J. Med. Sci. Sports.

[46] Kidokoro T., Shimizu Y., Edamoto K., Annear M. (2019). Classroom standing desks and time-series variation in sedentary behavior and physical activity among primary school children. Int. J. Environ. Res. Public Health.

[47] Egger F., Benzing V., Conzelmann A., Schmidt M. (2019). Boost your brain, while having a break! The effects of long-term cognitively engaging physical activity breaks on children’s executive functions and academic achievement. PLoS ONE.

[48] Schmidt M., Benzing V., Kamer M. (2016). Classroom-based physical activity breaks and children’s attention: Cognitive engagement works!. Front. Psychol..

[49] Ekanayake H.D.K., Salibi G., Tzenios N. (2023). Analysis of association between childhood overweight/obesity with screen time, sedentary life style and low levels of physical activity. Spec. J. Med. Acad. Other Life Sci..

[50] Curran F., Davis M.E., Murphy K., Tersigni N., King A., Ngo N., O’Donoghue G. (2023). Correlates of physical activity and sedentary behavior in adults living with overweight and obesity: A systematic review. Obes. Rev..

[51] Gonzalez Ramirez G., Bolaños Muñoz L. (2023). Relationship of Sedentary Lifestyle with Obesity and Comorbidities. Physical Activity and Bariatric Surgery.

[52] Poitras V.J., Gray C.E., Borghese M.M., Carson V., Chaput J.P., Janssen I., Katzmarzyk P.T., Pate R.R., Connor Gorber S., Kho M.E. (2016). Systematic review of the relationships between objectively measured physical activity and health indicators in school-aged children and youth. Appl. Physiol. Nutr. Metab. Physiol. Appl. Nutr. Metab..

[53] Greco G., Fischetti F., Cataldi C., Latino F. (2019). Effects of Shotokan Karate on resilience to bullying in adolescents. J. Hum. Sport Exerc..

[54] Mavilidi M.F., Drew R., Morgan P.J., Lubans D.R., Schmidt M., Riley N. (2020). Effects of different types of classroom physical activity breaks on children’s on-task behaviour, academic achievement and cognition. Acta Paediatr..

[55] Martin R., Murtagh E.M. (2016). Active Classrooms: A cluster randomised controlled trial evaluating the effects of a movement integration intervention on the physical activity levels of primary school children. J. Phys. Act. Health.

[56] McMullen J., Kulinna P., Cothran D. (2014). Physical activity opportunities during the school day: Classroom teachers’ perceptions of using activity breaks in the classroom. J. Teach. Phys. Educ..

[57] Norris E., van Steen T., Direito A., Stamatakis E. (2020). Physically active lessons in schools and their impact on physical activity, educational, health and cognition outcomes: A systematic review and meta-analysis. Br. J. Sports Med..

[58] Bobe G., Perera T., Frei S., Frei B. (2014). Brain breaks: Physical activity in the classroom for elementary school children. J. Nutr. Educ. Behav..

[59] Simon C., Schweitzer B., Oujaa M., Wagner A., Arveiler D., Triby E., Copin N., Blanc S., Platat C. (2008). Successful overweight prevention in adolescents by increasing physical activity: A 4-year randomized controlled intervention. Int. J. Obes..

[60] Li X.H., Lin S., Guo H., Huang Y., Wu L., Zhang Z., Ma J., Wang H.J. (2014). Effectiveness of a school-based physical activity intervention on obesity in school children: A nonrandomized controlled trial. BMC Public Health.

[61] Best J.R. (2010). Effects of Physical Activity on Children’s Executive Function: Contributions of Experimental Research on Aerobic Exercise. Dev. Rev. DR.

[62] Biederman J., Monuteaux M.C., Doyle A.E., Seidman L.J., Wilens T.E., Ferrero F., Morgan C.L., Faraone S.V. (2004). Impact of executive function deficits and attention-deficit/hyperactivity disorder (ADHD) on academic outcomes in children. J. Consult. Clin. Psychol..

[63] Budde H., Voelcker-Rehage C., Pietrabyk-Kendziorra S., Ribeiro P., Tidow G. (2008). Acute coordinative exercise improves attentional performance in adolescents. Neurosci. Lett..

[64] Dahlin K.I. (2010). Effects of working memory training on reading in children with special needs. Read. Writ..

[65] Davis C.L., Tomporowski P.D., McDowell J.E., Austin B.P., Miller P.H., Yanasak N.E., Allison J.D., Naglieri J.A. (2011). Exercise improves executive function and achievement and alters brain activation in overweight children: A randomized, controlled trial. Health Psychol..

[66] Castelli D.M., Hillman C.H., Buck S.M., Erwin H.E. (2007). Physical fitness and academic achievement in third- and fifth-grade students. J. Sport Exerc. Psychol..

[67] Dornhecker M., Blake J.J., Benden M., Zhao H., Wendel M. (2015). The effect of stand- biased desks on academic engagement: An exploratory study. Int. J. Health Promot. Educ..

[68] Hillman C., Pontifex M., Raine L., Castelli D., Hall E., Kramer A. (2009). The effect of acute treadmill walking on cognitive control and academic achievement in preadolescent children. Neuroscience.

[69] Luke S., Vail C.O., Ayres K.M. (2014). Using antecedent physical activity to increase on-task behavior in young children. Except. Child..

[70] Pontifex M.B., Saliba B.J., Raine L.B., Picchietti D.L., Hillman C.H. (2013). Exercise improves behavioral, neurocognitive, and scholastic performance in children with attention-deficit/hyperactivity disorder. J. Pediatr..

[71] Erickson K.I., Gildengers A.G., Butters M.A. (2013). Physical activity and brain plasticity in late adulthood. Dialogues Clin. Neurosci..

[72] van Praag H. (2008). Neurogenesis and exercise: Past and future directions. Neuromolecular Med..

[73] Donnelly J.E., Hillman C.H., Castelli D., Etnier J.L., Lee S., Tomporowski P., Lambourne K., Szabo-Reed A.N. (2016). Physical activity, fitness, cognitive function, and academic achievement in children: A systematic review. Med. Sci. Sports Exerc..

[74] de Greeff J.W., Bosker R.J., Oosterlaan J., Visscher C., Hartman E. (2018). Effects of physical activity on executive functions, attention and academic performance in preadolescent children: A meta-analysis. J. Sci. Med. Sport.

[75] Blom Skrade M.A. (2013). Integrated Classroom Physical Activity: Examining Perceived Need Satisfaction and Academic Performance in Children. Graduate Theses and Dissertations, Master’s Thesis.

[76] Resaland G.K., Aadland E., Moe V.F., Aadland K.N., Skrede T., Stavnsbo M., Suominen L., Steene-Johannessen J., Glosvik Ø., Andersen J.R. (2016). Effects of physical activity on schoolchildren’s academic performance: The Active Smarter Kids (ASK) cluster-randomized controlled trial. Prev. Med..

[77] Trudeau F., Shephard R.J. (2008). Physical education, school physical activity, school sports and academic performance. Int. J. Behav. Nutr. Phys. Act..

[78] Raiola G., Tafuri D., Altavilla G. (2015). Physical activity and its relation to body and ludic expression in childhood. Mediterr. J. Soc. Sci..

[79] Mazzeo F., Santamaria S., Monda V., Tafuri D., Dalia C. (2016). Dietary Supplements Use in Competitive and Non-Competitive Boxer: An Exploratory Study. Biol. Med..

[80] Monacis D., Colella D., Scarinci A. (2020). Health education intervention in primary school: Active breaks for the promotion of motor activity. Form@Re—Open J. Per La Form. Rete.

[81] Olivieri D. (2016). Mente-corpo, cervello, educazione: L’educazione fisica nell’ottica delle neuroscienze. Form. Insegn..

[82] Webster C.A., Russ L., Vazou S., Goh T.L., Erwin H. (2015). Integrating movement in academic classrooms: Understanding, applying and advancing the knowledge base. Obes. Rev..

[83] Di Palma D., Tafuri D. (2016). Special needs and inclusion in sport management: A specific literature review. Sport Sci..

[84] Schmidt M., Blum M., Valkanover S., Conzelmann A. (2015). Motor ability and self-esteem: The mediating role of physical self-concept and perceived social acceptance. Psychol. Sport Exerc..

[85] Christiansen L.B., Lund-Cramer P., Brondeel R., Smedegaard S., Holt A.-D., Skovgaard T. (2018). Improving children’s physical self-perception through a school-based physical activity intervention: The Move for Well-being in School study. Ment. Health Phys. Act..

[86] Latino F., Cataldi S., Fischetti F. (2021). Effects of an 8-Week Yoga-Based Physical Exercise Intervention on Teachers’ Burnout. Sustainability.

[87] Dobbins M., De Corby K., Robeson P., Husson H., Tirilis D. (2009). School-based physical activity programs for promoting physical activity and fitness in children and adolescents aged 6–18. Cochrane Database Syst. Rev..

[88] D’Anna C., Gomez Paloma F. (2019). La professionalità del docente di Educazione Fisica nella scuola primaria. Riflessioni, scenari attuali e prospettive. Ann. Online Didatt. Form. Docente.

[89] Mariani A.M., Piceci L., Melchiorri F. (2019). The contribution of physical activity on cognition and learning. Formazione&Insegnamento.

[90] Agrillo FD’Anna C., Gomez-Paloma F. (2012). Educazione motoria nella scuola primaria: Dalle neuroscienze alla prassi ducativa. Chinesiologia.

[91] Suglia A., Dettoni L. (2011). Un Modello di Progettazione in Scuola e Sicurezza: Dall’esperienza di un Lavoro in Rete Raccomandazioni Pratiche a Supporto della Progettazione.

[92] Colella D., Monacis D., Cinquepalmi D., D’Arando C. (2021). Interventions for the health promotion and motor activities in primary school. the sbam project! health, wellness, nutrition, movement at school. G. Ital. Educ. Alla Salut. Sport Didatt. Inclusiva Ital. J. Health Educ. Sports Incl. Didact..

[93] Errisuriz V.L., Golaszewski N.M., Born K., Bartholomew J.B. (2018). Systematic Review of Physical Education-Based Physical Activity Interventions Among Elementary School Children. J. Prim. Prev..

[94] Van Kann D., Kremers S., de Vries N.K., de Vries S.I., Jansen M. (2016). The effect of a school-centered multicomponent intervention on daily physical activity and sedentary behavior in primary school children: The Active Living study. Prev. Med..

[95] Sallis J.F., McKenzie T.L., Alcaraz J.E., Kolody B., Faucette N., Hovell M.F. (1997). The effect of a 2-year physical education program (SPARK) on physical activity and fitness in elementary school students. Am. J. Public Health.

[96] Luepker R.V., Perry C.L., McKinlay S.M., Nader P.R., Parcel G.S., Stone E.J., Webber L.S., Elder J.P., Feldman H.A., Johnson C.C. (1996). Outcomes of a field trial to improve children’s dietary patterns and physical activity. The Child and Adolescent Trial for Cardiovascular Health. CATCH collaborative group. JAMA.

[97] Simons-Morton B.G., Parcel G.S., Baranowski T., Forthofer R., O’Hara N.M. (1991). Promoting physical activity and a healthful diet among children: Results of a school-based intervention study. Am. J. Public Health.

[98] Caballero B., Clay T., Davis S., Ethelbah B., Holy Rock B., Lohman T., Norman J., Story M., Stone E., Stephenson L. (2003). Pathways Study Research Group, Pathways: A school-based, randomizzato controllato studio per la prevenzione dell’obesità negli scolari degli indiani d’America. Am. J. Clin. Nutr..

[99] Ronto R., Rathi N., Worsley A., Sanders T., Lonsdale C., Wolfenden L. (2020). Enablers and barriers to implementation of and compliance with school-based healthy food and beverage policies: A systematic literature review and meta-synthesis. Public Health Nutr..

[100] Masini A., Marini S., Leoni E., Lorusso G., Toselli S., Tessari A., Dallolio L. (2020). Active breaks: A pilot and feasibility study to evaluate the effectiveness of physical activity levels in a schoolbased intervention in an Italian primary school. Int. J. Environ. Res. Public Health.

[101] Latino F., Fischetti F., Colella D. (2020). The influence of physical activity on school-age children’s cognitive function and academic performance: A review of the literature. Form. Insegn..

[102] Colella D., Bellantonio S., D’Arando C., Monacis D. (2020). Interventi per la promozione delle attività motorie nella scuola primaria. Valutazione delle prestazioni motorie in relazione all’autoefficacia percepita ed al divertimento. Ital. J. Educ. Res..

[103] Centeio E.E., Brusseau T.A. (2024). The History of Physical Activity Promotion in Physical Education and Suggestions for Moving Forward. Kinesiol. Rev..

